# Specialized competency framework for pharmacists in managerial positions in sales and marketing (SCF-PMSM): development, validation, and correlates

**DOI:** 10.1186/s40545-023-00567-8

**Published:** 2023-05-11

**Authors:** Hala Sacre, Randa Aoun, Chadia Haddad, Carla Abou Selwan, Rony M. Zeenny, Marwan Akel, Aline Hajj, Carole Hassoun, Joya Namnoum, Katia Iskandar, Pascale Salameh

**Affiliations:** 1 INSPECT-LB (Institut National de Santé Publique, d’Épidémiologie Clinique et de Toxicologie-Liban), Beirut, Lebanon; 2 Research Department, Psychiatric Hospital of the Cross, P.O. Box 60096, Jal Eddib, Lebanon; 3 School of Health Sciences, Modern University for Business and Science, Beirut, Lebanon; 4 School of Medicine, Lebanese American University, Byblos, Lebanon; 5 SciencePRO Medical and Marketing Solutions, Jal Eddib, Lebanon; 6 Department of Pharmacy, American University of Beirut Medical Center, Beirut, Lebanon; 7 School of Pharmacy, Lebanese International University, Beirut, Lebanon; 8 School of Education, Lebanese International University, Beirut, Lebanon; 9 Laboratoire de Pharmacologie, Pharmacie Clinique et Contrôle de Qualité des Médicament (LPCQM), Faculty of Pharmacy, Saint Joseph University of Beirut, Beirut, Lebanon; 10 Faculté de Pharmacie, Université Laval, Québec, Canada; 11 Oncology Division, CHU de Québec Université Laval Research Center, Québec, Canada; 12 Roche Lebanon SARL, Beirut, Lebanon; 13 The Lebanon Pharma Group, Beirut, Lebanon; 14 Methodology and Statistics in Biomedical Research Unit, Faculty of Medicine, Paris-Saclay University, Kremlin-Bicêtre, Paris, France; 15 Faculty of Pharmacy, Lebanese University, Hadat, Lebanon; 16 Department of Primary Care and Population Health, University of Nicosia Medical School, 2417 Nicosia, Cyprus

**Keywords:** Competency framework, Pharmacist manager, Sales and marketing competency, Specialized competency

## Abstract

**Background:**

Pharmacists in sales and marketing roles need specific skills for managerial positions, and a framework for evaluating and developing these competencies is necessary. Currently, no such framework is known to exist.

**Objective:**

This study aimed to develop and validate a specialized competency framework for pharmacists in sales and marketing managerial positions and assess correlates related to the competency domains.

**Methods:**

This web-based study carried out between March and October 2022 enrolled a convenient sample of 60 pharmacists with managerial positions in sales and marketing, contacted by phone, working in the field of sales and marketing from the five governorates of Lebanon (Beirut, Beqaa, Mount Lebanon, South, and North).

**Results:**

The framework demonstrated good construct and structural validity in all domains except for emergency preparedness, which had a low correlation with other domains. Competencies were well correlated with respective domains, and behaviors had excellent loadings on corresponding competencies. As for the correlates of the competency domains, males were more confident than females (*p* < .05), and participants with more experience (or heavier workload) reported higher competency levels (*p* < .05), particularly for upper management skills, communication skills, and professional practice. Further, education level was not significantly correlated with declared competency, with experience being the most cited source of competence (68.25%), followed by postgraduate degrees (42.48%) and continuing education sessions (33.93%), while undergraduate education was the least reported (29.5%).

**Conclusion:**

This study could develop and validate the Specialized Competency Framework for Pharmacists in Managerial Positions in Sales and Marketing (SCF-PMSM) among a sample of Lebanese pharmacists. This framework demonstrated good reliability, content, construct, and structural validity in all the domains, with the competencies being well correlated with their respective domains and behaviors having excellent loadings on related competencies, except for emergency preparedness and response. It also revealed a mismatch between what is taught at undergraduate and postgraduate levels and the needs in practice.

**Supplementary Information:**

The online version contains supplementary material available at 10.1186/s40545-023-00567-8.

## Introduction

Pharmacists play a vital role in improving health outcomes by promoting the safe and efficient use of medications and contributing to building more efficient and sustainable health systems. They traditionally work in various settings, including the pharmaceutical industry sector, representing diverse scopes of practice opportunities [1–3].

Pharmacist can hold managerial positions in sales and marketing within pharmaceutical companies. Several factors can contribute to holding managerial positions, such as education, experience, leadership skills, industry knowledge, and networking. Other factors are not skill-related and include age and gender. Historically, managerial roles have been male-dominated, with women facing significant barriers to career advancement. In recent years, there has been a growing recognition of the value that diversity brings to organizational management, and efforts have been made to increase the representation of women in managerial positions. However, women remain underrepresented in management positions and continue to earn less than male managers [4]. Additionally, the age factor in managerial positions is complex and multifaceted. While age can contribute to effective leadership, it can become a barrier to equal opportunities for career advancement in some cultures [5]. Finally, the role of education in career attainment has long been debated, with diverging results regarding the importance of degrees in managerial success [6, 7].

Pharmacists in managerial positions are expected to have the necessary competencies to meet the job requirements. Competencies refer to the knowledge, skills, attitudes, and behaviors that affect an individual’s role or responsibilities related to job performance and are subject to improvement through training and development activities [8, 9]. Implementing a competency framework for pharmacy across different fields could lay the foundation for bridging the gap between traditional pharmacy education and the evolving needs of modern healthcare systems [10, 11].

In 2020, the International Pharmaceutical Federation (FIP) released a revised version of its Global Competency Framework [12], initially developed in 2012 [13], specifically intended to early-career pharmacists. This framework outlines competencies somewhat relevant to pharmacists with managerial positions in sales and marketing, particularly in the domains of Pharmaceutical Public Health Competencies, Organization and Management Competencies, and Professional/Personal Competencies (leadership and self-regulation). Nevertheless, these competencies are not sufficient since pharmacists working as sales and marketing managers are also required to demonstrate unique competencies specific to their roles in the industry, typically acquired with time and experience [14]. For example, in addition to the sales competencies (e.g., financial planning, business analytics, prospecting, upselling, time management, territory management, accountability and ownership, influencing skills, interpersonal skills, growth mindset, negotiation skills, digital engagement competencies and selling benefits), sales managers need to have four other critical competencies, i.e., coaching, mentorship, strategy, and advanced communication skills [15, 16]. As for marketing managers, they must have technical proficiency in marketing, including a deep understanding of common marketing models, strategies, and tools and the ability to analyze the market trends and market dynamics, the competitive landscape and behaviors and the marketplace factors. They should also demonstrate strong leadership, analytical skills, communication expertise, creativity [17], alongside strong business acumen. In 2020, the FIP released the Global Advanced Development Framework (GADF), which included competencies related to management. However, these competencies were not tailored for pharmacists in sales and marketing managerial positions [18].

While many educational programs are available, without a defined and recognized competency framework, it remains unclear whether these programs are relevant and beneficial. Furthermore, the World Health Organization (WHO) Global Strategy for the Healthcare Workforce 2030 highlights that health systems in any country can only function with a competent and accessible health workforce equipped with the necessary competencies to provide quality care [19]. Hence, pharmacists and stakeholders are encouraged to embrace and adopt competency-based education and training (CBET) in their professional development. CBET focuses on evaluating the performance of pharmacists in the workplace based on specific competencies and incorporating competency-based instructional approaches and assessments. It is also crucial to regularly revise and update these competencies to ensure their relevance and currency [10].

In view of all of the above, this study aimed to develop and validate the Specialized Competency Framework for Pharmacists in Managerial Positions in Sales and Marketing (SCF-PMSM) among a sample of Lebanese pharmacists and assess correlates related to the competency domains. The secondary objective was to compare education and gender.

## Methods

### Content validity: domains, competencies, and behaviors

After conducting a comprehensive literature review, a panel of seven experts, comprising two pharmaceutical company managers and five senior researchers involved in pharmacy education and academia, developed a specialized competency framework using the Delphi technique. Consensus was reached when an agreement of more than 90% was obtained. The framework agreed upon consisted of six domains. Domains 0 to 5 were adapted from several studies [20–22] and documented in a competency framework previously suggested by the Order of Pharmacists of Lebanon (OPL) [23] and inspired by the FIP Global Competency Framework [13]. Domain 0 included one set of competencies related to pharmaceutical knowledge. Domain 1 (Professional Communication) comprised four competencies, i.e., communication skills, negotiation skills, data processing and analysis skills, and information technology skills. Domain 2 (Organization & Management) covered two competencies, i.e., self-management and overall management. Domain 3 (Professional Practice) encompassed three competencies, i.e., standard practice, ethical practice, and legal practice. Domain 4 (Personal Practice) included two competencies: role modeling and teamwork. Domain 5 described upper management competencies in one set.

Furthermore, since the pandemic occurred after the previous framework was released by the OPL, a sixth domain related to Preparedness and Response to Emergency was added, inspired by several studies [11, 14, 24, 25]. It included four competencies: emergency preparedness and response, operation management (during emergencies), patient care and population health interventions, and evaluation, research, and dissemination for impact and outcomes.

### Study design

A cross-sectional study was carried out from March to October 2022 using an online questionnaire created on Google Forms for ease of distribution on social platforms (Facebook, Instagram, LinkedIn, and WhatsApp groups). The snowball technique was applied to reach pharmacists working as sales and marketing managers across the five Lebanese governorates (Beirut, Beqaa, Mount Lebanon, South Lebanon, and North Lebanon). Explanations about the topic and the different aspects of the study were available in the introductory section of the questionnaire. Respondents gave written consent before proceeding to the survey. Anonymity and confidentiality were ensured across the entire data collection process. All pharmacists registered with the OPL, holding managerial positions in sales and marketing, and living in Lebanon were eligible to participate.

### Ethical aspect

The Lebanese International University School of Pharmacy Research and Ethics committee approved the study protocol (2022RC-041-LIUSOP). This study was conducted in accordance with the ethical principles outlined in the Declaration of Helsinki.

### Sample size calculation

The minimum sample size was calculated using the CDC Epi-info software [26]. The expected frequency was set at 90% since specialized competencies and domains were expected to be fulfilled by working pharmacists. Accordingly, a minimum sample of 58 participants was required to produce an acceptable error of 5%, with a 95% confidence interval, a 5% alpha error, and a power of 80%.

### Questionnaire and variables

The questionnaire was in English, as this language is commonly spoken by healthcare professionals in Lebanon, and comprised two sections. The first section collected information related to sociodemographic features and professional status. In this part, participants were asked about their general sociodemographic data, including their age, gender, area of work, university of graduation, highest educational level, years of experience, the number of working hours per day, and the number of working days per week.

The second section consisted of the scale-based framework, which covered six domains, each comprising a set of competencies with their related behaviors (Additional file 1).

### Statistical analysis

The data were analyzed using SPSS software version 25. A descriptive analysis was done using the counts and percentages for categorical variables and means and standard deviations for continuous measures.

The content validity of the items was ensured by using a competency framework previously suggested by the Order of Pharmacists of Lebanon [23], with an additional domain related to emergency preparedness [11, 14, 24, 25]. An exploratory factor analysis using the principal component analysis technique was conducted for behaviors based on competencies and domains. For every analysis, the Kaiser–Meyer–Olkin (KMO) coefficient, Bartlett’s test for sphericity and total percentage of variance explained were reported. Cronbach’s alpha values were calculated for every competency to assess internal consistency (reliability). For structural validity, Pearson correlation coefficients were calculated to assess the correlation of the domains within their respective competencies, and their association with the other domains was used for convergent validity assessment.

Regarding the correlates of competencies, a multivariate analysis of covariance (MANCOVA) was carried out to compare the competencies domain between the highest degree (high academic degree vs. low) adjusted for age, gender, year of experience, number of working hours per day, university where they graduated as a pharmacist and level of education. Moreover, related multiple regressions were conducted to show the correlates of every domain. Stratifications over education level, gender, and experience duration were also presented; in the latter operation, polynomial contrasts were assessed to check the association of competencies with quartiles of experience duration. A *p*-value less than 0.05 was considered significant.

## Results

A total of 60 pharmacists with managerial positions in sales and marketing in Lebanon participated in the survey out of 101 initially approached (participation rate: 59.4%). Half of them were female (50%), the majority had a BS pharmacy degree (81.7%), used the English language in education (58.3%), worked in the Beirut area (65.0%), and did not have another field of work (76.7%). Also, 40% had a BS in pharmacy as the highest degree, and 36.7% and 28.3% graduated and earned their highest degree from the BAU, respectively. The average age of participants was 43.30 ± 9.58 years; the mean duration of work experience was 11.68 ± 8.86 years, the mean number of working days per week was 4.85 ± 1.23, and the mean number of working hours per day was 8.60 ± 3.74 (Table 1). On average, respondents reported having acquired these competencies mainly by experience (68%), followed by postgraduate studies (42%), continuing education sessions (34%), and undergraduate studies (30%) (Table 1).

**Table 1:** Sociodemographic and other characteristics of the pharmacists with managerial positions in sales and marketing (*N* = 60)

	Frequency (%)
*Gender*	
Male	30 (50.0%)
Female	30 (50.0%)
Education level*	
BS Pharmacy	49 (81.7%)
PharmD/DPharm	29 (48.3%)
Masters	28 (46.7%)
PhD	9 (15.0%)
*Highest degree related to your main field of work*	
BS Pharmacy	24 (40.0%)
Marketing	1 (1.7%)
Master’s degree	17 (28.3%)
MBA	1 (1.7%)
PharmD/DPharm	13 (21.7%)
PhD	3 (5.0%)
Residency and medical aesthetics sub specialty	1 (1.7%)
*Name of university graduated as a pharmacist*	
UL	7 (11.7%)
USJ	16 (26.7%)
AUB	1 (1.7%)
BAU	22 (36.7%)
LAU	7 (11.7%)
LIU	2 (3.3%)
Other	5 (8.2%)
*Name of university earning the highest degree from*	
UL	8 (13.3%)
USJ	9 (15.0%)
AUB	3 (5.0%)
BAU	17 (28.3%)
LAU	7 (11.7%)
Other	16 (26.7%)
*Language of pharmacy education*	
French	23 (38.3%)
English	35 (58.3%)
Both	1 (1.7%)
Other	1 (1.7%)
*Work location*	
Beirut	39 (65.0%)
Mont Lebanon	10 (16.7%)
North	4 (6.7%)
South	1 (1.7%)
Beqaa	1 (1.7%)
Currently not working	5 (8.3%)
*Having another field of work*	
I do not have another field of work	46 (76.7%)
Academia (teaching); Preceptor	7 (11.7%)
Managing director	1 (1.7%)
Regulatory	2 (3.4%)
Research	3 (5.0%)
Freelancer	1 (1.7%)
	*Mean ± SD*
*Age*	43.30 ± 9.58
*Number of working days per week*	4.85 ± 1.23
*Number of working hours per day*	8.60 ± 3.74
*Year of experience*	11.68 ± 8.86
*What percentage of these competencies did you learn?*	
During your undergraduate studies	29.50 (23.08)
During your postgraduate studies	42.48 (29.75)
During continuing education sessions	33.93 (29.02)
By experience	68.25 (22.47)

*AUB* American University of Beirut, *UL* Lebanese University, *USJ* Saint Joseph University of Beirut, *BAU* Beirut Arab University, *LAU* Lebanese American University, *LIU* Lebanese International University

### Factor analysis of competency domains

All competencies loaded on one factor and loaded adequately each on its respective domain (loading varied between 0.42 and 0.95). The percentage of explanation varied from 43.76% (management skills domain) to 92.00% (role modeling subdomain).

Cronbach’s alpha values were good to excellent, ranging from a minimum of 0.75 for the standard practice domain to a maximum of 0.96 for the upper management skills domain.

Table 2 presents the distribution of competencies according to the factor analysis.

**Table 2:** Factor analysis of the competencies of Lebanese pharmacists with managerial positions in sales and marketing (Promax rotated component matrix)

	Competency	Loading	Cronbach alpha
*Domain 0: Pharmaceutical knowledge*			
1	Answer questions of healthcare professionals on drugs/products and services (characteristics, contraindications, incremental benefits, etc.), as part of comprehensive patient care	0.914	0.913
2	Link scientific and medical knowledge to drug/product arguments	0.904	
3	Provide information on drugs/products and services and answer questions as part of therapeutic regimens associated with a pathology linked to the concerned drugs	0.854	
4	Exchange with healthcare professionals on scientific topics	0.847	
5	Maintain and develop product knowledge through training	0.791	
6	Have thorough knowledge of the different categories of pharmaceuticals, and the therapeutic value of each drug category	0.774	
Kaiser–Meyer–Olkin (KMO) 0.889, Bartlett’s test of sphericity < 0.001, Percentage of variance explained 72.05%			
*Domain 1: Professional communication skills*			
	1.1.Communication		
1	Summarize the key elements involved in medical/marketing communication in the healthcare environment	0.915	0.923
2	Use effective verbal, non-verbal, listening, and written communication skills to communicate accurately and appropriately	0.905	
3	Communicate effectively with physicians, other healthcare professionals, support staff, and relevant third parties	0.896	
4	Use the information, arguments, business aids developed by the pharmaceutical company	0.894	
5	Explain the characteristics and the proper use of drugs/products based on the needs of healthcare professionals and market demands	0.829	
6	Use appropriate language and checks comprehension	0.801	
7	Display knowledge of pharmaceuticals during sales presentations to doctors and other healthcare professionals	0.799	
8	Demonstrate respect, cultural awareness, sensitivity, and empathy when communicating	0.717	
9	Take ownership of the content of the information prepared by the scientists responsible for the pharmaceutical company	0.429	
Kaiser–Meyer–Olkin (KMO) 0.835, Bartlett’s test of sphericity < 0.001, Percentage of variance explained 65.81%			
	1.2.Negotiation		
1	Analyze the call/visit (SWOC analysis) and plan the next step	0.853	0.937
2	Conclude the call/visit & prepare reports	0.845	
3	Demonstrate knowledge of sales techniques	0.833	
4	Process requests for information and objections	0.831	
5	Identify/address the healthcare professionals’ concerns/needs and their patient care practices by using appropriate probing/questioning	0.828	
6	Apply active listening techniques with the healthcare professional	0.824	
7	Adapt to different communication styles	0.823	
8	Establish a quality relationship with healthcare professionals	0.805	
9	Animate professional communication gatherings and develop long-term professional relationships/partnerships with healthcare professionals	0.748	
Note: Kaiser–Meyer–Olkin (KMO) 0.861, Bartlett’s test of sphericity < 0.001, Percentage of variance explained 67.48%			
	1.3.Data processing analysis skills		
1	Monitor actions and professional communication during visits	0.852	0.858
2	Collect, analyze and transmit questions to the concerned departments of the company	0.826	
3	Collect and process information on drugs/products, from documentation and training sessions to prepare for visits and communication actions	0.822	
4	Collect and transmit pharmacovigilance information	0.802	
5	Appraise the commercial competitor environment when evaluating the opportunity for new medicine under development or a currently marketed product	0.794	
6	Describe the commercial healthcare environment in which pharmaceutical medicine operates	0.498	
7	Apply competitive intelligence and report information to its hierarchy	0.494	
Kaiser–Meyer–Olkin (KMO) 0.662, Bartlett’s test of sphericity < 0.001, Percentage of variance explained 55.00%			
	1.4.Information technology		
1	Master research of information via electronic databases	0.885	0.850
2	Inform and update files	0.867	
3	Save and transmit calls/visits reports to the company database	0.850	
4	Optimize the use of computerized/electronic devices to prepare presentations, reports, charts, and manage business and information processing	0.731	
Kaiser–Meyer–Olkin (KMO) 0.713, Bartlett’s test of sphericity < 0.001, Percentage of variance explained 69.82%			
*Domain 2: Organization and Management Skills*			
	2.1. Self-management skills		
1	Engage in regular professional development activities	0.863	0.922
2	Demonstrate organization and efficiency in carrying out the work	0.850	
3	Ensure work time and processes are planned and managed appropriately	0.825	
4	Demonstrate the ability to prioritize work appropriately	0.825	
5	Take responsibility as appropriate in the workplace	0.820	
6	Organize visits according to the predefined objectives and through teamwork	0.780	
7	Reflect on and demonstrate learning from critical incidents	0.728	
8	Engage in professional organization activities	0.727	
9	Ensure punctuality and reliability	0.687	
Kaiser–Meyer–Olkin (KMO) 0.861, Bartlett’s test of sphericity < 0.001, Percentage of variance explained 62.65%			
	2.2. Management skills		
1	Describe the pharmaceutical industry (internal environment, structure and function, key stakeholders and commercial drivers) and explain how these business elements impact on the broader healthcare market place	0.773	0.831
2	Demonstrate an understanding of the principles of organization and management	0.763	
3	Provide regular feedback on the drugs/products and the market	0.712	
4	Convey any helpful information from the market with all the company's concerned people/departments (medical representatives, direct manager, product manager, medical manager, medical science liaison, CRA, etc.)	0.678	
5	Work effectively with the company hierarchy	0.652	
6	Apply the national and international code of ethics guidelines when organizing any of the scientific events mentioned above	0.612	
7	Work effectively with the documented procedures and policies within the workplace	0.597	
8	Apply the company's compliance, procedures, and safety rules (road, IT, etc.)	0.594	
9	Organize round tables, expert meetings, advisory boards, lectures, CME conferences, staff meetings, awareness campaigns, and others in coordination with different departments within the company and service providers	0.531	
Kaiser–Meyer–Olkin (KMO) 0.466, Bartlett’s test of sphericity < 0.001, Percentage of variance explained 43.76%			
*Domain 3: Professional practice*			
	3.1. Standard practice		
1	Take responsibility for their own actions	0.776	0.755
2	Demonstrate awareness of the position of trust of the profession and practice in a manner that upholds that trust	0.747	
3	Maintain a consistently high standard of work	0.713	
4	Carry out duties as a medical representative in a professional manner	0.710	
5	Recognize their scope of practice and the extent of their current competency and expertise and works accordingly	0.707	
6	Treat others with sensitivity, empathy, respect, and dignity	0.679	
Kaiser–Meyer–Olkin (KMO) 0.706, Bartlett’s test of sphericity < 0.001, Percentage of variance explained 52.23%			
	3.2. Ethical practice		
1	Recognize ethical dilemmas in practice scenarios and reason through dilemmas in a structured manner	0.855	0.802
2	Understand obligations under the principles of the statutory Code of Conduct for Pharmacists and act accordingly	0.844	
3	Make and justify decisions in a manner that reflects the statutory Code of Conduct for pharmacists and pharmacy law	0.827	
4	Implement standard operating procedures and Code of Ethics	0.679	
Kaiser–Meyer–Olkin (KMO) 0.618, Bartlett’s test of sphericity < 0.001, Percentage of variance explained 64.70%			
	3.3. Legal practice		
1	Demonstrate an awareness of and adheres to professional indemnity requirements	0.844	0.889
2	Identify laws and regulations related to sales and marketing practices	0.804	
3	Drive up pharmacovigilance information by following the internal procedures and regulations	0.794	
4	Use and take into account the drug-related pharmaceutical and economic regulation and its evolution to inform and answer questions from healthcare professionals	0.789	
5	Use tools related to the product (summary of product characteristics, product file, transparency commission opinion, validated data, etc.)	0.779	
6	Integrate into business the rules of advertising, promotion, distribution, and delivery of the drug and their changes	0.775	
7	Raise awareness and provide information on regulatory changes	0.670	
Kaiser–Meyer–Olkin (KMO) 0.654, Bartlett’s test of sphericity < 0.001, Percentage of variance explained 60.97%			
*Domain 4: Personal skills*			
	4.1. Role modelling		
1	Inspire confidence and apply assertiveness skills as appropriate	0.866	0.840
2	Build credibility and portray the profession in a positive light by being professional and well-informed	0.856	
3	Have effective leadership skills	0.828	
4	Contribute to the initiation, development, and continuous improvement of business plans	0.743	
Kaiser–Meyer–Olkin (KMO) 0.720, Bartlett’s test of sphericity < 0.001, Percentage of variance explained 67.98%			
	4.2. Team working skills		
1	Recognize when it is appropriate to seek advice from experienced colleagues, refer decisions to a higher level of authority, or include other colleagues in the decision	0.959	0.913
2	Recognize the value of transversal teamwork	0.959	
Kaiser–Meyer–Olkin (KMO) 0.500, Bartlett’s test of sphericity < 0.001, Percentage of variance explained 92.00%			
*Domain 5: Upper management skills*			
1	Integrate into business the rules of advertising, promotion, distribution and delivery of the drug and their changes	0.890	0.962
2	Explain his/her accountability to key stakeholders, society and the profession of pharmaceutical medicine	0.810	
3	Reflect on and demonstrate learning from critical incidents	0.808	
4	Contribute to the initiation, development and continuous improvement of business plan	0.794	
5	Apply quality and performance improvement concepts to address organizational performance issues	0.792	
6	Use tools related to the product (SPC, product file, Transparency Commission opinion, validated data, etc.)	0.791	
7	Ensure organizational excellence by developing critical evaluation skills, encouraging improvement and innovation in managing change	0.785	
8	Inform and update files	0.764	
9	Use and take into account the drug related pharmaceutical and economic regulation and its evolution to inform and answer questions from healthcare professionals	0.739	
10	Ensure that the knowledge, skills and behaviors associated with the competent practice of pharmaceutical medicine are communicated effectively, using the best techniques and practices whilst participating in the education of colleagues and stakeholders	0.731	
11	Support the success of the organization by actively contributing to develop strategic plans to achieve goals, manage resources and people, and leverage performance	0.720	
12	Build credibility and portrays the profession in a positive light by being professional and well informed	0.720	
13	Identify strengths, deficiencies and limits in one's knowledge and expertise	0.718	
14	Demonstrate the ability to prioritize work appropriately	0.704	
15	Ensure punctuality and reliability	0.699	
16	Raise awareness and provide information on regulatory changes	0.694	
17	Ensure work time and processes are appropriately planned and managed	0.688	
18	Drive up pharmacovigilance information by following the internal procedures and regulations	0.684	
19	Inspire confidence and applies assertiveness skills as appropriate	0.683	
20	Optimize the use of computerized/electronic devices to prepare presentations, reports, charts, etc., and manage business and information processing	0.677	
21	Demonstrate organization and efficiency in carrying out the work	0.675	
22	Organize visits according to the predefined objectives and through teamwork	0.668	
23	Recognize the value of transversal teamwork	0.654	
24	Master research of information via electronic databases	0.629	
25	Describe the principles and practices of people management and leadership to apply them within their own working environment; sets learning and improvement goals	0.621	
26	Recognize when it is appropriate to seek advice from experienced colleagues, refer decisions to a higher level of authority or to include other colleagues in the decision	0.575	
27	Take responsibility as appropriate in the workplace	0.570	
28	Save and transmit calls/visits reports to the company database	0.539	
29	Organize networks and build and maintain relationships, encouraging contribution and working with interprofessional teams to meet the business objectives	0.537	
30	Manage of prescribers/customers files	0.521	
31	Work effectively as a member or leader of a healthcare team or other professional groups	0.520	
Kaiser–Meyer–Olkin (KMO) 0.617, Bartlett’s test of sphericity < 0.001, Percentage of variance explained 48.49%			
*Domain 6: Pharmacist Preparedness and Response in Emergency Situations*			
	6.1. Emergency Preparedness and Response		
1	Partner with local authorities	0.827	0.875
2	Balance stockpile and availability of drugs for existing/chronic conditions	0.808	
3	Address medication shortage and mitigation plan	0.806	
4	Check for training opportunities	0.733	
5	Follow actions and recommendations of local authorities	0.707	
6	Involve trainees and staff in emergency response	0.698	
7	Check for FDA/EMA Emergency Use Authorizations (EUAs) and expedited review and approval of tests/drugs for treatment	0.679	
8	Check for volunteering opportunities	0.633	
Kaiser–Meyer–Olkin (KMO) 0.751, Bartlett’s test of sphericity < 0.001, Percentage of variance explained 54.66%			
	6.2. Operation Management		
1	Secure PPEs or other needed materials	0.871	0.928
2	Secure sanitizers and other medications when needed	0.867	
3	Participate in interdisciplinary training to EPR teams	0.856	
4	Develop workplace training and safety protocols (e.g., social distancing)	0.821	
5	Procure essential medications and supplies	0.805	
6	Ensure medication delivery/safe storage	0.779	
7	Monitor workers/assistants for symptoms	0.776	
8	Adapt working hours to meet essential services during crises	0.772	
Kaiser–Meyer–Olkin (KMO) 0.808, Bartlett’s test of sphericity < 0.001, Percentage of variance explained 67.11%			
	6.3. Patient Care and Population Health Interventions		
1	Identify at-risk populations	0.924	0.887
2	Manage panic buying	0.913	
3	Answer EPR-related calls	0.876	
Kaiser–Meyer–Olkin (KMO) 0.732, Bartlett’s test of sphericity < 0.001, Percentage of variance explained 81.87%			
	6.4. Evaluation, Research, and Dissemination for Impact and Outcomes		
1	Participate in research and studies on EPR	0.920	0.900
2	Publish and/or disseminate findings	0.909	
3	Combat misinformation by disseminating evidence-based information to patients and sharing it on social media	0.896	
4	Develop training programs for peers and other healthcare workers	0.781	
	Kaiser–Meyer–Olkin (KMO) 0.745, Bartlett’s test of sphericity < 0.001, Percentage of variance explained 77.09%		

### Structural and convergent validity

All competencies were correlated together, except for the Preparedness and Response in the Emergency Situations competency, which was correlated only with the professional communication competency. The correlation coefficients varied between weak (0.255) for Preparedness and Response in Emergency Situations and Professional Communication Skills and very strong (0.824) for Upper Management Skills and Organization and Management Skills.

Moreover, all behaviors correlated well with their respective competencies. The correlation values for the first competency varied from moderate (r = 0.42) to very strong (r = 0.90). In the second competency, the correlation values were strong (> 0.6). For Competency 3, the correlation values varied from moderate (r = 0.50) to strong (r = 0.87). In Competency 4, a weak correlation (r = 0.33) was found between Role Modeling and Team Working domains. In competency 6, the correlation varied from moderate (r = 0.45) to strong (r = 0.90) (Table 3).

**Table 3:** Correlation analysis between the domains and competencies

Correlation analysis between the main domains						
	Domain 1	Domain 2	Domain 3	Domain 4	Domain 5	Domain 6
Domain 0	0.673^***^	0.601^***^	0.488^***^	0.580^***^	0.591^***^	0.215
Domain 1	–	0.851^***^	0.684^***^	0.712^***^	0.776^***^	0.255^*^
Domain 2		–	0.790^***^	0.816^***^	0.824^***^	0.247
Domain 3			–	0.839^***^	0.801^***^	0.202
Domain 4				–	0.798^***^	0.223
Domain 5					–	0.190
Domain 6						–
Domain 0: Pharmaceutical knowledge; Domain 1: Professional communication; Domain 2: Organization & management; Domain 3: Professional Practice; Domain 4: Personal Practice; Domain 5: Upper management; Domain 6: Preparedness and Response to Emergency						

Correlation analysis of the competencies of domain 1: professional communication				
	Competency 1.1	Competency 1.2	Competency 1.3	Competency 1.4
Domain 1	0.904***	0.856***	0.791***	0.739***
Competency 1.1	–	0.700***	0.572***	0.665***
Competency 1.2		–	0.598***	0.472***
Competency 1.3			–	0.424**
Competency 1.4				–
Competency 1.1: Communication skills; Competency 1.2: Negotiation skills; Competency 1.3; Data processing and analysis skills; Competency 1.4: Information technology skills				

Correlation analysis of the competencies of Domain 2: Organization and Management		
	Competency 2.1	Competency 2.2
Domain 2	0.907***	0.895***
Competency 2.1: Self-management	–	0.623***
Competency 2.2: Overall management		–

Correlation analysis of the competencies of Domain 3: Professional practice			
	Competency 3.1	Competency 3.2	Competency 3.3
Domain 3	0.848***	0.789***	0.874***
Competency 3.1: Standard practice	–	0.692***	0.526***
Competency 3.2: Ethical practice		–	0.508***
Competency 3.3: Legal practice			–

Correlation analysis of the Competencies of Domain 4: personal practice		
	Competency 4.1	Competency 4.2
Domain 4	0.909***	0.700***
Competency 4.1: Role modeling	–	0.339**
Competency 4.2: Teamworking		–

Correlation analysis of competencies of Domain 6: preparedness and emergency response				
	Competency 6.1	Competency 6.2	Competency 6.3	Competency 6.4
Domain 6	0.849***	0.901***	0.858***	0.730***
Competency 6.1: Emergency Preparedness and Response		0.691***	0.582***	0.455***
Competency 6.2: Operation Management (during emergencies)			0.777***	0.483***
Competency 6.3: Patient care & population health interventions				0.633***
Competency 6.4: Evaluation, Research, and Dissemination for Impact and Outcomes				-

*** < 0.001; ** < 0.01; * < 0.05

### Multivariable analysis

The MANCOVA analysis was performed taking the competency domains as the dependent variables and the highest academic degree (vs. the lowest) as the independent variable after adjusting for the covariates (age, gender, year of experience, number of working hours per day, and the university of pharmacy education) (Table 4).

**Table 4:** Multivariable analysis of covariance (MANCOVA)

	Beta	*P* value	Confidence interval	
			Lower	Upper
*Domain 0: pharmaceutical knowledge*				
Age	0.007	0.929	− 0.148	0.162
Gender (female vs male*)	0.047	0.966	− 2.174	2.269
Year of experience	0.115	0.156	− 0.046	0.276
Number of working hours per day	0.208	0.124	− 0.059	0.475
university graduated as a pharmacist (UL vs other*)	2.336	0.206	− 1.327	5.999
university graduated as a pharmacist (USJ vs other*)	1.040	0.458	− 1.754	3.834
university graduated as a pharmacist (BAU vs other*)	1.826	0.146	− 0.658	4.310
Level of education BS (yes vs no*)	0.722	0.632	− 2.285	3.729
Level of education PhD (yes vs no*)	− 0.724	0.609	− 3.554	2.105
Highest degree (PhD, Masters, MBA vs PharmD, BS*)	0.437	0.703	− 1.856	2.730
*Domain 1: professional communication skills*				
Age	− 0.131	0.580	− 0.606	0.343
Gender (female vs male*)	− 4.381	0.203	− 11.202	2.440
Year of experience	0.261	0.293	− 0.232	0.754
Number of working hours per day	0.645	0.120	− 0.175	1.466
university graduated as a pharmacist (UL vs other*)	1.351	0.810	− 9.896	12.598
university graduated as a pharmacist (USJ vs other*)	− 1.940	0.652	− 10.519	6.640
university graduated as a pharmacist (BAU vs other*)	− 2.210	0.563	− 9.838	5.419
Level of education BS (yes vs no*)	2.392	0.605	− 6.841	11.625
Level of education PhD (yes vs no*)	− 3.461	0.427	− 12.149	5.227
Highest degree (PhD, Masters, MBA vs PharmD, BS*)	− 3.139	0.375	− 10.179	3.901
*Domain 2: organization and management skills*				
Age	− 0.141	0.277	− 0.398	0.117
Gender (female vs male*)	− **4.952**	**0.010**	− **8.649**	− **1.255**
Year of experience	0.176	0.191	− 0.091	0.443
Number of working hours per day	**0.433**	**0.056**	− **0.011**	**0.878**
university graduated as a pharmacist (UL vs other*)	2.785	0.363	− 3.311	8.881
university graduated as a pharmacist (USJ vs other*)	− 0.354	0.879	− 5.004	4.296
university graduated as a pharmacist (BAU vs other*)	− 3.126	0.135	− 7.260	1.009
Level of education BS (yes vs no*)	3.383	0.181	− 1.622	8.387
Level of education PhD (yes vs no*)	− 3.005	0.206	− 7.715	1.704
Highest degree (PhD, Masters, MBA vs PharmD, BS*)	− 2.153	0.262	− 5.969	1.663
*Domain 3: professional practice*				
Age	− 0.075	0.445	− 0.271	0.121
Gender (female vs male*)	− **3.187**	**0.028**	− **6.005**	− **0.368**
Year of experience	0.027	0.788	− 0.176	0.231
Number of working hours per day	**0.498**	**0.005**	**0.159**	**0.837**
university graduated as a pharmacist (UL vs other*)	3.215	0.171	− 1.433	7.863
university graduated as a pharmacist (USJ vs other*)	0.513	0.772	− 3.033	4.058
university graduated as a pharmacist (BAU vs other*)	− 1.339	0.398	− 4.491	1.814
Level of education BS (yes vs no*)	**5.042**	**0.011**	**1.226**	**8.857**
Level of education PhD (yes vs no*)	− 1.658	0.358	− 5.249	1.933
Highest degree (PhD, Masters, MBA vs PharmD, BS*)	− 1.423	0.331	− 4.332	1.487
*Domain 4: personal practice*				
Age	− 0.022	0.522	− 0.089	0.046
Gender (female vs male*)	− **1.006**	**0.042**	− **1.973**	− **0.038**
Year of experience	0.030	0.399	− 0.040	0.100
Number of working hours per day	0.113	0.058	− 0.004	0.229
university graduated as a pharmacist (UL vs other*)	1.106	0.170	− 0.489	2.702
university graduated as a pharmacist (USJ vs other*)	− 0.402	0.510	− 1.619	0.815
university graduated as a pharmacist (BAU vs other*)	− 0.622	0.254	− 1.704	0.460
Level of education BS (yes vs no*)	1.146	0.085	− 0.164	2.456
Level of education PhD (yes vs no*)	− 0.787	0.206	− 2.019	0.446
Highest degree (PhD, Masters, MBA vs PharmD, BS*)	− 0.164	0.743	− 1.163	0.835
*Domain 5: upper management*				
Age	− 0.220	0.244	− 0.595	0.155
Gender (female vs male*)	− 2.791	0.303	− 8.176	2.595
Year of experience	**0.355**	**0.073**	− **0.034**	**0.744**
Number of working hours per day	0.385	0.238	− 0.263	1.033
university graduated as a pharmacist (UL vs other*)	3.493	0.433	− 5.386	12.373
university graduated as a pharmacist (USJ vs other*)	− 2.768	0.415	− 9.542	4.005
university graduated as a pharmacist (BAU vs other*)	− 2.833	0.349	− 8.856	3.190
Level of education BS (yes vs no*)	4.244	0.248	− 3.045	11.534
Level of education PhD (yes vs no*)	− 1.714	0.618	− 8.573	5.146
Highest degree (PhD, Masters, MBA vs PharmD, BS*)	− 1.356	0.626	− 6.914	4.202
*Domain 6: preparedness and emergency response*				
Age	0.011	0.970	− 0.573	0.595
Gender (female vs male*)	− 1.558	0.711	− 9.945	6.830
Year of experience	− 0.299	0.327	− 0.905	0.308
Number of working hours per day	**1.062**	**0.040**	**0.053**	**2.071**
university graduated as a pharmacist (UL vs other*)	2.235	0.747	− 11.595	16.065
university graduated as a pharmacist (USJ vs other*)	− 7.781	0.145	− 18.331	2.769
university graduated as a pharmacist (BAU vs other*)	1.997	0.671	− 7.383	11.378
Level of education BS (yes vs no*)	10.933	0.059	− 0.421	22.287
Level of education PhD (yes vs no*)	9.078	0.094	− 1.606	19.761
Highest degree (PhD, Masters, MBA vs PharmD, BS*)	0.457	0.916	− 8.200	9.114

In the global model, the independent variable is highest degree. Covariates are: age, gender, year of experience, number of working hours per day, university graduated as a pharmacist and level of education

*AUB* American University of Beirut, *UL* Lebanese University, *USJ* Saint Joseph University of Beirut, *BAU* Beirut Arab University, *LAU* Lebanese American University, *LIU* Lebanese International University

*Reference group

Considering the organization and management skills domain as the dependent variable, being a female (Beta = − 4.95) was significantly associated with lower competencies. Taking the professional practice as the dependent variable, the results showed that being a male (Beta = − 3.18), having a BS degree (Beta = 5.04), and working for longer hours per day (Beta = 0.49) were significantly associated with higher competencies. Considering the personal skills domain, the results showed that being a female (Beta = − 1.00) was significantly associated with lower competencies.

As for the Preparedness and Response in Emergency Situations domain, a higher number of working hours per day (Beta = 1.06) was significantly associated with higher competencies.

No significant association was found between the independent variables used and pharmaceutical knowledge, professional communication skills, and upper management skills (*p* > 0.05 for all). Only experience showed a borderline association with upper management skills (*p* = 0.07).

### Stratification over education

**Fig. 1:**
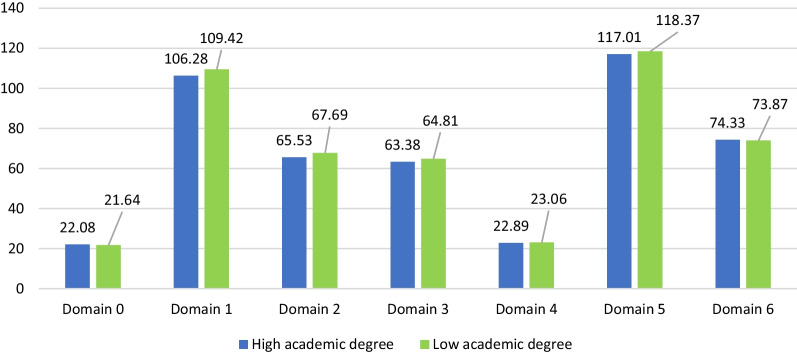
Mean values of the domain scoring by highest degree (high academic degree vs low) adjusted for age, gender, year of experience, number of working hours per day, and university of graduation as a pharmacist (*p* > 0.05 for all). Domain 0: Pharmaceutical knowledge; Domain 1: Professional communication; Domain 2: Organization and management; Domain 3: Professional Practice; Domain 4: Personal Practice; Domain 5: Upper management; Domain 6: Preparedness and Response to Emergency

Figure 1 shows the means of the competency domain scoring between the highest academic degree (vs. the lowest) after adjustment over age, gender, year of experience, number of working hours per day, and the university of pharmacy education. No significant difference was found for all the competencies comparing pharmacists with higher and lower academic degrees (*p* > 0.05 for all).

### Stratification over gender

**Fig. 2:**
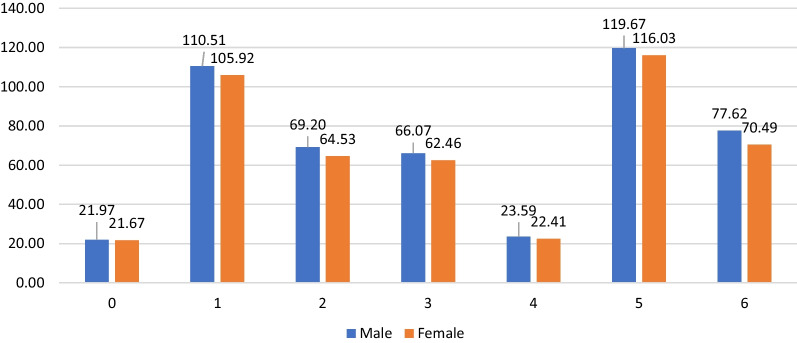
Mean values of the domain by gender, adjusted for age, highest degree, year of experience, number of working hours per day, and university of graduation as a pharmacist. Domain 0: Pharmaceutical knowledge; Domain 1: Professional communication; Domain 2: Organization and management; Domain 3: Professional Practice; Domain 4: Personal Practice; Domain 5: Upper management; Domain 6: Preparedness and Response to Emergency. P-values were: Domain 0: 0.113; Domain 1: 0.007; Domain 2: 0.006; Domain 3: 0.009; Domain 4:0.066; Domain 5: 0.745; Domain 6: 0.131

The means of the competencies’ domain scoring between males and females, after adjustment over age, year of experience, number of working hours per day, university of graduation as a pharmacist, and level of education are shown in Fig. 2. Males declared significantly higher confidence than females in domains 1 (Professional communication), 2 (Organization and management), 3 (Professional Practice), and 4 (Personal Practice; borderline significance). In other domains, the difference was also in favor of males, but did not reach statistical significance (Fig. 2).

### Stratification over experience

**Fig. 3:**
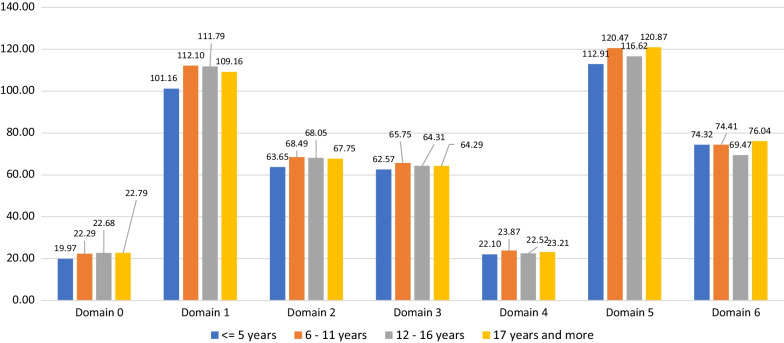
Mean values of the domain scoring by experience quartile, adjusted for age, gender, highest degree, number of working hours per day, and university of graduation as a pharmacist. Domain 0: Pharmaceutical knowledge; Domain 1: Professional communication; Domain 2: Organization & management; Domain 3: Professional Practice; Domain 4: Personal Practice; Domain 5: Upper management; Domain 6: Preparedness and Response to Emergency. P-values for polynomial contrasts were: Domain 0: 0.132; Domain 1: 0.013; Domain 2: 0.114; Domain 3: 0.282; Domain 4:0.009; Domain 5: 0.051; Domain 6: 0.775

Work experience duration was associated with higher competency scores for all domains; it reached statistical significance for domains 1 (professional communication), 4 (personal practice), and 5 (upper management) (Fig. 3).

## Discussion

To our knowledge, this study is the first to develop and validate the Specialized Competency Framework for Pharmacists in Managerial Positions in Sales and Marketing (SCF-PMSM) among a sample of Lebanese pharmacists; hence, it might be challenging to make comparisons with previous studies. This framework demonstrated good reliability, content, construct, and structural validity in all the domains, with the competencies being well correlated with their respective domains and behaviors having excellent loadings on related competencies, except for emergency preparedness and response. Expectedly, this domain had a low correlation with others (significance was only shown with the professional communication domain) since the concept is not taught at any level of the pharmacy curricula. Further, no studies were found about emergency preparedness among pharmacists with managerial positions in sales and marketing. However, this lack of preparedness was reported in various health sectors during the COVID-19 pandemic [27]. Despite the statement of policy released by the FIP in 2017 and published frameworks related to emergency preparedness, no countries seem to have included a part related to this aspect in their frameworks [11, 14]. Furthermore, comparisons were made with findings from other sectors due to the lack of data and specificity in the literature on the competencies of pharmacists with managerial positions in sales and marketing. Mapping our framework with the management and leadership areas of the FIP-GADF would help optimize the suggested framework [18].

As for the correlates of the competency domains, gender and years of experience were the main factors associated with self-declared confidence in different behaviors. Consistent with previous literature findings, males were more confident than females in the following domains: Professional Communication, Organization & Management, Professional Practice, and Personal Practice. The gender gap in confidence has long been described as the rule rather than the exception in different sectors (medicine, business, and finance) and age groups (schoolchildren, young adults, and adults) and seems to be cross-cultural [28–31]. In a sample of about one million people from 48 countries [32], self-esteem among men was significantly higher in each culture.

Furthermore, participants with longer experience (or heavier workload) declared having higher levels of competency, particularly for the subdomains of upper management skills, communication skills, and professional practice. Studies have shown that education level does not necessarily correlate with declared competency in the workforce, as people with the same degree can have different levels of competency. Further, competency acquisition is a lifelong process that continues after graduation. The on-the-job experience was the most significant predictor of competency, rather than formal education [33–35].

Also, education level was not significantly correlated with declared competency; this result was confirmed by the fact that experience was the most cited source of competence, followed by postgraduate degrees and continuing education sessions, while undergraduate education was the least reported. A study exploring self-reported competency of graduating nursing students and their perception of the quality of the undergraduate program revealed that some competency domain scores were significantly associated with previous professional experience, but none were associated with degree grades [33].

These results suggest a mismatch between what is taught at undergraduate and postgraduate levels and the needs in actual practice, confirming previous findings among pharmacists from different professional sectors in Lebanon [23, 36]. This discrepancy described in the literature triggered a reflection on studies and strategies to counter this issue and reduce the gap between formal education and job market needs [37, 38].

In light of all these facts, relevant stakeholders should place more emphasis on work experience and continuing professional development rather than solely relying on formal education as an indicator of competency.

### Limitations and strengths

This study has several limitations. It was conducted online, and a selection bias may have occurred due to the lengthy questionnaire. Additionally, the study relied on self-reported data, and participants may have overestimated or underestimated their competencies, and recall bias is also possible due to the economic crisis affecting the healthcare sector. The study may also have residual confounding bias and a low power due to the small sample size. Therefore, further research on a larger scale is recommended to address these limitations.

Despite these limitations, developing and implementing a framework to assess competencies among pharmacists with managerial positions in sales and marketing is crucial for improving pharmacy practice in Lebanon. This framework can align with international pharmacy standards, adapt to local needs, and guide universities and pharmaceutical companies in identifying gaps and developing expertise in the sales and marketing field.

## Conclusion

This study could develop and validate the Specialized Competency Framework for Pharmacists in Managerial Positions in Sales and Marketing (SCF-PMSM) among a sample of Lebanese pharmacists. This framework demonstrated good reliability, content, construct, and structural validity in all the domains, with the competencies being well correlated with their respective domains and behaviors having excellent loadings on related competencies, except for emergency preparedness and response. It also revealed a mismatch between what is taught at undergraduate and postgraduate levels and the needs in practice, confirming previous findings among pharmacists from different professional sectors in Lebanon. Hence, relevant stakeholders should place more emphasis on work experience and continuing professional development rather than solely relying on formal education as an indicator of competency to reduce the gap between formal education and job market needs.

## Supplementary Information

**Additional file 1**. Advanced Competencies for Sales and Marketing Manager Pharmacists questionnaire.

## Data Availability

The datasets generated and/or analyzed during the current study are available from the corresponding author upon reasonable request.

## Abbreviations

FIP: International Pharmaceutical Federation
WHO: World Health Organization
CBET: Competency-based education and training
OPL: Order of Pharmacists of Lebanon
SPSS: Statistical Package for the Social Sciences
KMO: Kaiser–Meyer–Olkin
MANCOVA: Multivariate analysis of covariance
AUB: American University of Beirut
UL: Lebanese University
USJ: Saint Joseph University of Beirut
BAU: Beirut Arab University
LAU: Lebanese American University
LIU: Lebanese International University
