# Supplementary material for: Specialized competency framework for pharmacists in managerial positions in sales and marketing (SCF-PMSM): development, validation, and correlates
Source: J Pharm Policy Pract. 2023 May 11;16:64. doi: 10.1186/s40545-023-00567-8 (PMC10173228; doi:10.1186/s40545-023-00567-8)
Supplement: Supplementary file 1 — Additional file 1. Advanced Competencies for Sales and Marketing Manager Pharmacists questionnaire. [file 40545_2023_567_MOESM1_ESM.pdf]

Advanced Competencies for Sales and Marketing Manager Pharmacists

Dear pharmacist,

You are invited to participate in a survey about advanced competencies and skills acquired upon graduation of your highest degree related to your current field of work.

This study conducted by a group of academic researchers aims to determine the domains that need strengthening for an optimal-performing public health system.

Your participation in this study is voluntary and anonymous, and the information gathered in this 20-minute questionnaire will be treated confidentially. By completing it, you are consenting to participate in this study.

We thank you in advance for your time,

The research team.

Informed consent

Please check all the boxes to proceed to the survey

☐ I have read and understood the above information

☐ I understand that my participation is voluntary

☐ I understand that my data will be kept confidential

☐ I agree to participate in this study

DEMOGRAPHICS

1. **Age:**
2. **Gender:** ☐M ☐F
3. **Level of education:**
   ☐BS Pharmacy ☐PharmD/DPharm ☐Masters ☐PhD ☐Other:
4. **Highest degree related to your main field of work:**
   ☐BS Pharmacy ☐PharmD/DPharm ☐Masters ☐PhD ☐Other:
5. **Year of graduation from school/faculty of pharmacy:**
6. **University you graduated from as a pharmacist:**
   ☐UL ☐USJ ☐BAU ☐LAU ☐LIU ☐Other, country:
7. **University you earned your highest degree from:**
   ☐UL ☐USJ ☐BAU ☐LAU ☐AUB ☐LIU ☐Other, country:
8. **Language of pharmacy education:**
   ☐French ☐English ☐Other:
9. **Work Location:**
   ☐Beirut ☐Mount Lebanon ☐North Lebanon ☐South Lebanon ☐Beqaa
   ☐Currently not working
10. **Number of working days per week:**
11. **Number of working hours per day:**
12. **How long have you been practicing as a marketing/sales manager?**
13. **Do you have another field of work? (Please select all that apply):**
    ☐I do not have another field of work
    ☐Academia (teaching)
    ☐Preceptor
    ☐Clinical pharmacy
    ☐Research
    ☐Other:

SALES & MARKETING MANAGER PHARMACIST COMPETENCIES

QUESTION: How confident are you in applying the list of sales & marketing competencies?	Very confident	Fairly confident	Neither/ I don't know	Slightly confident	Not confident at all
**0 Pharmaceutical Knowledge**	**0.1 Pharmaceutical Knowledge**				
**0.1.1** Have thorough knowledge of the different categories of pharmaceuticals, and the therapeutic value of each drug category.					
**0.1.2** Provide information on drugs/products and services and answer questions as part of therapeutic regimens associated with a pathology linked to the concerned drugs.					
**0.1.3** Answer questions of healthcare professionals on drugs/products and services (characteristics, contraindications, incremental benefits, etc.), as part of comprehensive patient care.					
**0.1.4** Link scientific and medical knowledge to drug/product arguments.					
**0.1.5** Exchange with healthcare professionals on scientific topics.					
**0.1.6** Maintain and develop product knowledge through training.					
**1 Professional Communication Skills**	**1.1 Communication**				
**1.1.1** Display knowledge of pharmaceuticals during sales presentations to doctors and other healthcare professionals.					
**1.1.2** Summarize the key elements involved in medical/marketing communication in the healthcare environment.					
**1.1.3** Explain the characteristics and the proper use of drugs/products based on the needs of healthcare professionals and market demands.					
**1.1.4** Take ownership of the content of the information prepared by the scientists responsible for the pharmaceutical company.					
**1.1.5** Use the information, arguments, business aids developed by the pharmaceutical company.					
**1.1.6** Use effective verbal, non-verbal, listening, and written communication skills to communicate accurately and appropriately.					
**1.1.7** Communicate effectively with physicians, other healthcare professionals, support staff, and relevant third parties.					
**1.1.8** Use appropriate language and checks comprehension.					
**1.1.9** Demonstrate respect, cultural awareness, sensitivity, and empathy when communicating.					
**1 Professional Communication Skills**	**1.2 Negotiation**				
**1.2.1** Establish a quality relationship with healthcare professionals.					
**1.2.2** Identify/address the healthcare professionals' concerns/needs and their patient care practices by using appropriate probing/questioning.					
**1.2.3** Apply active listening techniques with the healthcare professional.					

**1.2.4** Demonstrate knowledge of sales techniques.					
**1.2.5** Adapt to different communication styles.					
**1.2.6** Process requests for information and objections.					
**1.2.7** Conclude the call/visit & prepare reports.					
**1.2.8** Analyze the call/visit (SWOC analysis) and plan the next step.					
**1.2.9** Animate professional communication gatherings and develop long-term professional relationships/partnerships with healthcare professionals.					
**1 Professional Communication Skills**	**1.3 Data Processing Analysis Skills**				
**1.3.1** Collect and process information on drugs/products, from documentation and training sessions to prepare for visits and communication actions.					
**1.3.2** Collect, analyze and transmit questions to the concerned departments of the company.					
**1.3.3** Collect and transmit pharmacovigilance information.					
**1.3.4** Describe the commercial healthcare environment in which pharmaceutical medicine operates.					
**1.3.5** Appraise the commercial competitor environment when evaluating the opportunity for new medicine under development or a currently marketed product.					
**1.3.6** Apply competitive intelligence and report information to its hierarchy.					
**1.3.7** Monitor actions and professional communication during visits.					
**1 Professional Communication Skills**	**1.4 Information Technology**				
**1.4.1** Save and transmit calls/visits reports to the company database.					
**1.4.2** Inform and update files.					
**1.4.3** Master research of information via electronic databases.					
**1.4.4** Optimize the use of computerized/electronic devices to prepare presentations, reports, charts, and manage business and information processing.					
**1.4.5** Manage prescribers' files.					
**2 Organization and Management Skills**	**2.1 Self-Management Skills**				
**2.1.1** Demonstrate organization and efficiency in carrying out the work.					
**2.1.2** Organize visits according to the predefined objectives and through teamwork.					
**2.1.3** Ensure work time and processes are planned and managed appropriately.					
**2.1.4** Demonstrate the ability to prioritize work appropriately.					
**2.1.5** Take responsibility as appropriate in the workplace.					
**2.1.6** Ensure punctuality and reliability.					
**2.1.7** Reflect on and demonstrate learning from critical incidents.					
**2.1.8** Engage in regular professional development activities					
**2.1.9** Engage in professional organization activities					
**2 Organization and Management Skills**	**2.2 Management Skills**				
**2.2.1** Describe the pharmaceutical industry (internal environment, structure and function, key stakeholders and commercial drivers) and explain how these business elements impact on the broader healthcare market place.					

**2.2.2** Demonstrate an understanding of the principles of organization and management.					
**2.2.3** Work effectively with the documented procedures and policies within the workplace.					
**2.2.4** Work effectively with the company hierarchy.					
**2.2.5** Provide regular feedback on the drugs/products and the market.					
**2.2.6** Convey any helpful information from the market with all the company's concerned people/departments (medical representatives, direct manager, product manager, medical manager, medical science liaison, CRA, etc.).					
**2.2.7** Apply the company's compliance, procedures, and safety rules (road, IT, etc.).					
**2.2.8** Organize round tables, expert meetings, advisory boards, lectures, CME conferences, staff meetings, awareness campaigns, and others in coordination with different departments within the company and service providers.					
**2.2.9** Apply the national and international code of ethics guidelines when organizing any of the scientific events mentioned above.					
**3 Professional Practice**	**3.1 Standard Practice**				
**3.1.1** Carry out duties as a medical representative in a professional manner.					
**3.1.2** Demonstrate awareness of the position of trust of the profession and practice in a manner that upholds that trust.					
**3.1.3** Treat others with sensitivity, empathy, respect, and dignity.					
**3.1.4** Take responsibility for their own actions.					
**3.1.5** Recognize their scope of practice and the extent of their current competency and expertise and works accordingly.					
**3.1.6** Maintain a consistently high standard of work.					
**3 Professional Practice**	**3.2 Ethical Practice**				
**3.2.1** Understand obligations under the principles of the statutory Code of Conduct for Pharmacists and act accordingly.					
**3.2.2** Make and justify decisions in a manner that reflects the statutory Code of Conduct for pharmacists and pharmacy law.					
**3.2.3** Recognize ethical dilemmas in practice scenarios and reason through dilemmas in a structured manner.					
**3.2.4** Implement standard operating procedures and Code of Ethics.					
**3 Professional Practice**	**3.3 Legal Practice**				
**3.3.1** Identify laws and regulations related to sales and marketing practices.					
**3.3.2** Demonstrate an awareness of and adheres to professional indemnity requirements.					
**3.3.3** Use and take into account the drug-related pharmaceutical and economic regulation and its evolution to inform and answer questions from healthcare professionals.					
**3.3.4** Integrate into business the rules of advertising, promotion, distribution, and delivery of the drug and their changes.					
**3.3.5** Raise awareness and provide information on regulatory changes.					

**3.3.6** Use tools related to the product (summary of product characteristics, product file, transparency commission opinion, validated data, etc.).					
**3.3.7** Drive up pharmacovigilance information by following the internal procedures and regulations.					
**4 Personal Skills**	**4.1 Role Modeling**				
**4.1.1** Inspire confidence and apply assertiveness skills as appropriate.					
**4.1.2** Build credibility and portray the profession in a positive light by being professional and well-informed.					
**4.1.3** Contribute to the initiation, development, and continuous improvement of business plans.					
**4.1.4** Have effective leadership skills.					
**4 Personal Skills**	**4.2 Team Working Skills**				
**4.2.1** Recognize the value of transversal teamwork.					
**4.2.2** Recognize when it is appropriate to seek advice from experienced colleagues, refer decisions to a higher level of authority, or include other colleagues in the decision.					
**5 Upper Management Skills**					
**5.1** Describe the principles and practices of people management and leadership to apply them within their own working environment; sets learning and improvement goals.					
**5.2** Ensure that the knowledge, skills and behaviors associated with the competent practice of pharmaceutical medicine are communicated effectively, using the best techniques and practices whilst participating in the education of colleagues and stakeholders.					
**5.3** Identify strengths, deficiencies, and limits in one’s knowledge and expertise.					
**5.4** Organize networks and build and maintain relationships, encouraging contribution and working with interprofessional teams to meet the business objectives.					
**5.5** Support the success of the organization by actively contributing to develop strategic plans to achieve goals, manage resources and people, and leverage performance.					
**5.6** Contribute to the initiation, development and continuous improvement of business plan.					
**5.7** Ensure organizational excellence by developing critical evaluation skills, encouraging improvement and innovation in managing change.					
**5.8** Work effectively as a member or leader of a healthcare team or other professional groups.					
**5.9** Explain his/her accountability to key stakeholders, society and the profession of pharmaceutical medicine.					
**5.10** Save and transmit calls/visits reports to the company database.					
**5.11** Inform and update files.					
**5.12** Master research of information via electronic databases.					
**5.13** Optimize the use of computerized/electronic devices to prepare presentations, reports, charts, etc., and manage business and information processing.					
**5.14** Manage of prescribers/customers files.					

**5.15** Recognize the value of transversal teamwork.					
**5.16** Recognize when it is appropriate to seek advice from experienced colleagues, refer decisions to a higher level of authority or to include other colleagues in the decision.					
**5.17** Inspire confidence and applies assertiveness skills as appropriate.					
**5.18** Build credibility and portrays the profession in a positive light by being professional and well informed.					
**5.19** Use and take into account the drug related pharmaceutical and economic regulation and its evolution to inform and answer questions from healthcare professionals.					
**5.20** Integrate into business the rules of advertising, promotion, distribution and delivery of the drug and their changes.					
**5.21** Raise awareness and provide information on regulatory changes.					
**5.22** Use tools related to the product (SPC, product file, Transparency Commission opinion, validated data, etc.).					
**5.23** Drive up pharmacovigilance information by following the internal procedures and regulations.					
**5.24** Apply quality and performance improvement concepts to address organizational performance issues.					
**5.25** Demonstrate organization and efficiency in carrying out the work.					
**5.26** Organize visits according to the predefined objectives and through teamwork.					
**5.27** Ensure work time and processes are appropriately planned and managed.					
**5.28** Demonstrate the ability to prioritize work appropriately.					
**5.29** Take responsibility as appropriate in the workplace.					
**5.30** Ensure punctuality and reliability.					
**5.31** Reflect on and demonstrate learning from critical incidents.					
**6 Pharmacist Emergency Preparedness and Response (EPR)**	**6.1 Emergency Preparedness and Response**				
**6.1.1** Check for volunteering opportunities					
**6.1.2** Check for training opportunities					
**6.1.3** Address medication shortage and mitigation plan					
**6.1.4** Balance stockpile and availability of drugs for existing/chronic conditions					
**6.1.5** Partner with local authorities					
**6.1.6** Check for FDA/EMA Emergency Use Authorizations (EUAs) and expedited review and approval of tests/drugs for treatment					
**6.1.7** Follow actions and recommendations of local authorities					
**6 Pharmacist Preparedness and Response in Emergency Situations**	**6.2 Operations Management**				
**6.2.1** Procure essential medications and supplies					
**6.2.2** Ensure medication delivery/safe storage					
**6.2.3** Develop workplace training and safety protocols (e.g., social distancing)					
**6.2.4** Secure PPEs or other needed materials, when applicable					
**6.2.5** Monitor workers/assistants for symptoms					
**6.2.6** Adapt working hours to meet essential services during crises					

**6.2.7** Secure sanitizers and other medications when needed					
**6.2.8** Participate in interdisciplinary training to EPR teams					
**6 Pharmacist Preparedness and Response in Emergency Situations**	**6.3 Patient Care and Population Health Interventions**				
**5.3.7** Manage panic buying					
**5.3.9** Answer EPR-related calls					
**6 Pharmacist Preparedness and Response in Emergency Situations**	**6.4 Evaluation, Research, and Dissemination for Impact and Outcomes**				
**6.4.1** Participate in research and studies on EPR					
**6.4.2** Publish and/or disseminate findings					
**6.4.3** Combat misinformation by disseminating evidence-based information to patients and sharing it on social media					
**6.4.4** Develop training programs to peers and other healthcare workers					

14. **What percentage of these competencies did you acquire during your undergraduate studies?**

15. **What percentage of these competencies did you acquire during your postgraduate studies?**

16. **What percentage of these competencies did you acquire during from continuing education sessions?**

17. **What percentage of these competencies did you acquire by experience?**
